# The relationship between members of the canonical NF-κB pathway, components of tumour microenvironment and survival in patients with invasive ductal breast cancer

**DOI:** 10.18632/oncotarget.16031

**Published:** 2017-03-09

**Authors:** Lindsay Bennett, Elizabeth A. Mallon, Paul G. Horgan, Andrew Paul, Donald C. McMillan, Joanne Edwards

**Affiliations:** ^1^ Wolfson Wohl Cancer Research Centre, Institute of Cancer Sciences, College of Medical, Veterinary and Life Sciences, University of Glasgow, Glasgow, Scotland, United Kingdom; ^2^ Department of Pathology, Queen Elizabeth University Hospital, Glasgow, Scotland, United Kingdom; ^3^ Academic Unit of Surgery, School of Medicine, University of Glasgow, Glasgow Royal Infirmary, Glasgow, Scotland, United Kingdom; ^4^ Strathclyde Institute of Pharmacy and Biomedical Sciences, University of Strathclyde, Glasgow, Scotland, United Kingdom

**Keywords:** breast cancer, NF-κB, tumour microenvironment, survival

## Abstract

The aim of the present study was to examine the relationship between tumour NF-κB activation, tumour microenvironment including local inflammatory response (LIR) and cancer-specific survival in patients with operable ductal breast cancer.

Immunohistochemistry (tissue microarray of 376 patients) and western blotting (MCF7 and MDA-MB-231 breast cancer cells) was performed to assess expression of key members of the canonical NF-κB pathway (inhibitory kappa B kinase (IKKβ) and phosphorylated p65 Ser-536 (p-p65)). Following silencing of IKKβ, cell viability and apoptosis was assessed in both MCF7 and MDA-MB-231 cell lines.

P-p65 was associated with cancer-specific survival (CSS) (nuclear P=0.042 and total P=0.025). High total p-p65 was associated with increase grade tumour grade (P=0.010), ER positivity (P=0.023), molecular subtype (P=0.005), lower Klintrup-Makinen grade (P=0.013) and decreased CD138 count (P=0.032). On multivariate analysis, total p-p65 expression independently associated with poorer CSS (P=0.020). *In vitro* work demonstrated that the canonical NF-κB pathway was inducible by exposure to TNFα in ER-positive MCF7 cells and to a lesser extent in ER-negative MDA-MB-231 cells. Reduction of IKKβ expression by siRNA transfection increased levels of apoptosis and reduced cell viability in both MCF7 (P=<0.001, P=<0.001, respectively) and MDA-MB-231 cells (P=>0.001, P=0.002, respectively). This is consistent with the hypothesis that canonical IKKβ-NF-κB signalling drives tumour survival.

These results suggest that activation of the canonical NF-κB pathway is an important determinant of poor outcome in patients with invasive ductal breast cancer.

## INTRODUCTION

Breast cancer is the most common female cancer in the UK and, despite earlier detection and improved treatments, remains the second most common cause of cancer death in women [1, 2]. Therefore, it is clear that a further understanding of this disease process is required.

For many years an association between cancer and inflammation has been suspected and evidence has been gathered over the last decade to support this relationship [3, 4]. However, little is known about the signalling pathways that link these processes. The canonical mammalian Nuclear Factor kappa B (NF-κB) pathway regulates genes involved in many of the processes considered as hallmarks of cancer including inflammation, proliferation and apoptosis [5, 6]. It is therefore not surprising that NF-κB has been hypothesised to provide the link between inflammation and cancer [6–9].

Aberrant activity of various NF-κB components has been observed in a number of solid tumours including colorectal [10] and prostate cancer [11–13]. In breast cancer, increased NF-κB activity has been observed in rat mammary tumours compared to normal tissue [14] and numerous studies employing cell lines have reported an association with NF-κB activity and endocrine resistance [15]. In human breast cancer specimens, expression of the different NF-κB subunits have been observed [14, 15]. Levels of phosphorylated p65 have been reported to correlate with HER2 expression, tumour size, grade and presence of metastases in a cohort of 57 patients [15] and high DNA binding activity of p50 was associated with decreased disease free survival in a cohort of 81 cases [17]. In a larger cohort of 208 cases, p65 expression was not associated with disease specific survival but was associated with luminal B subtype [16]. A clear consensus of the prognostic power of members of the canonical NF-κB pathway has not been reached.

Therefore the aims of the present study are to examine the relationship between key members of the canonical NF-κB pathway (IKKα and p65 (RelA)), local inflammatory infiltrate and survival in a cohort of patients with primary operable invasive ductal breast cancer.

## RESULTS

A total of 376 patients who presented with invasive ductal breast cancer were included in the study. Seventy three patients had local or distant recurrences, the median follow-up of survivors was 164 months with 79 cancer-associated deaths and 79 non-cancer deaths. ER, PR, HER2 and Ki67 status was available for these patients allowing us to determine molecular subtype: 44% of patients had Luminal A disease (ER and PR positive, HER2 negative and low Ki67 <14%), 21% had Luminal B disease (ER, PR and HER2 positive or ER, PR and high Ki67 (>14%)), 21% had triple negative disease (ER, PR and HER2 negative) and 12% with HER2 enriched disease (ER and PR negative, HER2 positive). We were unable to define molecular subtype in 2% of cases due to missing data.

**Figure 1:**
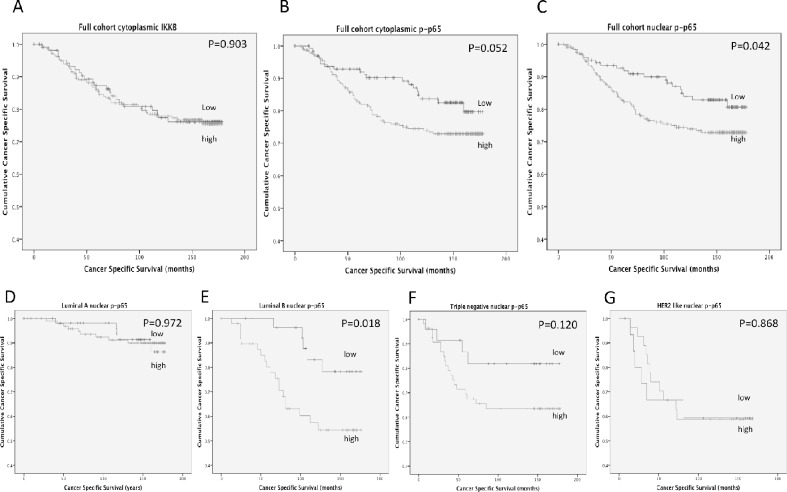
**(A)** shows the relationship between IKKβ expression and cancer-specific survival in patients with primary operable invasive ductal breast cancer (P=0.903). **(B)** shows the relationship between cytoplasmic p-p65 expression and cancer-specific survival in patients with primary operable invasive ductal breast cancer (P=0.052). **(C)** shows the relationship between nuclear p-p65 expression and cancer-specific survival in patients with invasive ductal breast cancer (P=0.042). **(D)** shows the relationship between nuclear p-p65 expression and cancer-specific survival in patients with Luminal A invasive ductal breast cancer (P=0.972). **(E)** shows the relationship between nuclear p-p65 expression and cancer-specific survival in patients with Luminal B invasive ductal breast cancer (P=0.018). **(F)** shows the relationship between nuclear p-p65 expression and cancer-specific survival in patients with triple negative invasive ductal breast cancer (P=0.120). **(G)** shows the relationship between nuclear p-p65 expression and cancer-specific survival in patients with HER2 enriched invasive ductal breast cancer (P=0.868).

Expression of IKKβ was observed in the cytoplasm and ranged from 0-275 weighed histoscore units (Supplementary Figure 1). P-p65 expression was observed in the cytoplasm ranging from 0-220 histoscore units and the nucleus ranging from 0-160 histoscore units (Supplementary Figure 2). Expression was split into tertiles, the first tertile was considered low expression and the second and third tertiles were grouped to provide a measure of moderate/high expression. Expression of IKKβ was not associated with cancer-specific survival (Figure 1A) (P=0.903), cytoplasmic p-p65 expression showed a non-significant trend towards shorter cancer-specific survival (Figure 1B, Table 2) (P=0.052) and nuclear p-p65 expression showed a significant association with shorter cancer-specific survival (Figure 1C) (P=0.042). For nuclear p-p65 10 year cancer-specific survival was stratified from 84% to 71% (P=0.022).

When patients were subdivided by molecular subtype, no significant associations were observed between IKKβ or cytoplasmic p-p65 expression in any of the molecular subtypes. When nuclear p-p65 was stratified by molecular subtype (Figure 1D-1G) the only subtype where the association with cancer-specific survival was upheld was in patients with Luminal B disease (P=0.018) (Figure 1E), 10 year cancer-specific survival was stratified from 83% to 57% (P=0.006). A trend towards cancer-specific survival was observed in the triple negative subgroup, however due to low patient numbers this did not reach significance (P=0.120). No association between nuclear p-p65 and cancer-specific survival was observed in patients with Luminal A disease (Figure 1D) (P=0.972) or HER2 enriched disease (Figure 1G) (P=0.868).

As nuclear p-p65 was associated with cancer-specific survival, the relationship between this and tumour characteristics was subsequently examined (Table 1). High expression of nuclear p-p65 was associated with a high tumour grade (P=0.04) and tumour recurrence (P=0.008), but not tumour size, ER status, HER2 status or molecular subtype (Table 1). When associations with the tumour microenvironment were investigated, nuclear p-p65 expression was not significantly associated with tumour budding, T-lymphocytes or Klintrup-Makinen grade. However an association was observed with increasing tumour stromal percentage (P=0.033) (Table 1).

**Table 1:** The relationship between clinicopathological characteristics and nuclear p-p65 expression in patients with invasive ductal breast cancer

Patients n=376	low(n=126)	high(n=250)	*P*
Age (≤50/ >50 years)	36/90	72/178	0.963
Size (≤20/ 21-50/ >50 mm)	71/51/3	142/102/6	1.000
Grade (I / II / III)	27/56/43	40/97/113	**0.040**
Involved lymph node (negative/positive)	62/64	134/111	0.186
ER status (no/yes)	44/82	89/161	0.495
PR status (no/yes)	70/56	133/117	0.374
HER2 status (no/ yes)	100/23	200/49	0.470
Molecular subtype (Luminal A/Luminal B/triple negative/HER2 enriched)	54/29/25/15	111/50/55/29	0.897
Tumour necrosis (low/high)	54/69	105/140	0.486
Lymph vessel invasion (no/yes)	85/41	160/89	0.309
Blood vessel invasion (no/yes)	108/18	221/28	0.246
Klintrup–Mäkinen grade (weak/strong)	89/34	174/71	0.444
CD68+ (low/moderate/high)	34/45/46	61/95/93	0.705
CD4+ (low/moderate/high)	52/25/48	99/58/92	0.968
CD8+ (low/moderate/high)	26/47/52	73/92/84	0.058
CD138+ (low/moderate/high)	72/14/39	129/35/85	0.382
Angiogenesis (low/moderate/high)	37/44/42	78/81/82	0.789
Tumour stroma percentage (low/high)	82/42	138/110	**0.033**
Tumour budding (low/high)	79/47	162/87	0.367
Locoregional treatment (lumpectomy+radiotherapy/mastectomy+ radiotherapy)	39/87	97/153	0.083
Systemic treatment (hormonal/hormonal+ chemotherapy/chemotherapy/ none)	66/28/25/6	125/51/58/11	0.641
Recurrence (no recurrence/recurrence)	104/20	180/69	**0.008**

Bold indicates a significant association. Number of patients with missing data is not displayed, when values do not give a sum of 100% this is due to data being unavailable.

When nuclear p-p65 was entered into a multivariate model using a backwards conditional method with clinicopathological parameters, nuclear p-p65 expression was not independently associated with cancer-specific survival in the full cohort (Table 2) but was independently associated with cancer-specific survival in Luminal B disease (P=0.019, HR 1.95 (1.12-3.41)).

**Table 2:** The relationship between clinicopathological characteristics, nuclear p-p65 and cancer-specific survival in patients with invasive ductal breast cancer

	Univariate analysis		Multivariate analysis Nuclear p-p65		Multivariate analysisTotal tumour cell p-p65	
Patients (n=376)	Hazard ratio(95% CI)	P value	Hazard ratio(95% CI)	P value	Hazard ratio(95% CI)	P value
Age (≤50/ >50 years)	1.33(0.812-2.24)	0.248				
Size (≤20/ 21-50/ >50 mm)	1.72(1.16-2.56)	0.007				
Grade (I / II / III)	1.94(1.37-2.75)	<0.001				
Involved lymph node (no/yes)	3.34(2.04-5.47)	<0.001	2.39(1.40-4.07)	0.001	2.13(1.23-3.68)	0.007
ER status (no/yes)	0.46(0.29-0.72)	0.001				
PR status (no/yes)	0.37(0.22-0.61)	<0.001	0.43(0.25-0.730)	0.002	0.44(0.25-0.74)	0.002
HER2 status (no/ yes)	2.15(1.33-3.48)	0.002				
Tumour necrosis (low/high)	4.33(2.41-7.94)	<0.001	3.71 (1.97-6.97)	<0.001	3.51(1.90-6.74)	<0.001
Lymph vessel invasion (no/yes)	3.59(2.28-5.66)	<0.001				
Blood vessel invasion (no/yes)	3.01(1.79-5.06)	<0.001	2.42(1.38-4.24)	0.002	2.19(1.23-3.90)	0.007
Klintrup–Mäkinen grade (week/strong)	1.32(0.82-2.13)	0.249				
CD68+ (low/moderate/high)	0.78(0.59-1.04)	0.093				
CD4+ (low/moderate/high)	1.00(0.782-1.28)	0.938				
CD8+ (low/moderate/high)	0.62(0.46-0.82)	0.001	0.54(0.40-0.74)	<0.001	0.54(0.40-0.72)	<0.001
CD138+ (low/moderate/high)	1.36(1.07-1.73)	0.01	1.461.11-1.91)	0.005	1.41(1.09-1.87)	0.009
Tumour stroma percentage (low/high)	1.26(1.44-3.54)	<0.001	2.12(1.32-3.41)	0.002	2.10(1.30-3.38)	0.002
Tumour budding (low/high)	2.45(1.57-3.82)	<0.001	2.12(1.31-3.42)	0.002	1.79(1.07-2.97)	0.025
Nuclear p-p65 (low/high)	1.29(1.00-1.67)	0.045	1.31(0.99-1.73)	0.057	NA	NA
Total Tumour cell p-p65 (both low/ one high)	1.21(1.02-1.44)	0.029	NA	NA	1.25(1.03-1.53)	0.024

**Figure 2:**
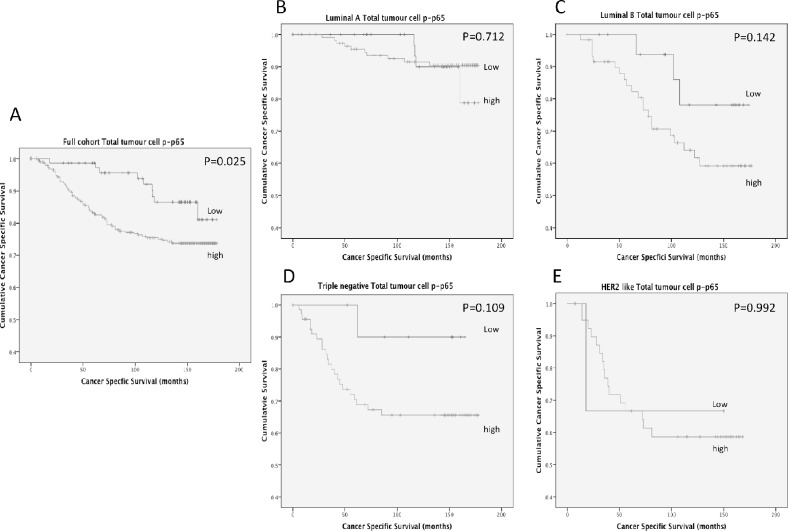
**(A)** shows the relationship between total tumour cell p-p65 expression and cancer-specific survival in patients with primary operable invasive ductal breast cancer (P=0.025). **(B)** shows the relationship between total tumour cell p-p65 expression and cancer-specific survival in patients with Luminal A invasive ductal breast cancer (P=0.712). **(C)** shows the relationship between total tumour cell p-p65 expression and cancer-specific survival in patients with Luminal B invasive ductal breast cancer (P=0.142). **(D)** shows the relationship between total tumour cell p-p65 expression and cancer-specific survival in patients with Triple negative invasive ductal breast cancer (P=0.109). **(E)** shows the relationship between total tumour cell p-p65 expression and cancer-specific survival in patients with HER2 enriched invasive ductal breast cancer (P=0.992).

To examine the relationship of cancer-specific survival and total tumour cell p-p65, a cumulative prognostic score of cytoplasmic and nuclear p-p65 was examined. Patients with both low cytoplasmic and nuclear expression were classified as the low expression group and patients with either moderate/high cytoplasmic or nuclear expression were classified as the moderate/high expression group. Moderate/high expression of total tumour cell expression of p-p65 was significantly association with shorter cancer-specific survival (Figure 2A) (P=0.025) and 10 year cancer-specific survival was stratified from 86% (low expression) to 75% (moderate/high expression) (P=0.006). When total tumour cell p-p65 was stratified by molecular subtype (Figure 2B-2E) the association between total tumour cell p-p65 and cancer-specific survival was lost in patients with Luminal A disease (Figure 2B)(P=0.712) (10 year survival, 90% vrs 92%, P=0.930) and in patients with HER2 enriched disease (Figure 2E) (P=0.992) (10 year survival, 67% vrs 59%, P=0.865). However a trend towards cancer-specific survival was observed in patients with Luminal B disease (Figure 2C)(P=0.142) (10 year survival, 78% vrs 55%, P=0.091) and in triple negative disease (Figure 2D) (P=0.109) (10 year survival, 90% vrs 66%, P=0.071), however due to low patient numbers these did not reach significance.

As total tumour cell p-p65 was associated with cancer-specific survival, the relationship between this prognostic model and tumour characteristics was subsequently examined (Table 3). High expression of total tumour cell expression of p-p65 was associated with a high tumour grade (P=0.01), ER positive disease (P=0.023), molecular subtype (P=0.005) and tumour recurrence (P=0.001). When associations with the tumour microenvironment were investigated, high total tumour cell expression was not significantly associated with tumour stromal percentage, tumour budding or T-lymphocytes. However an association was observed with local inflammatory cell infiltrate as assessed by Klintrup-Makinen grade (P=0.013) and density of CD138^+^ B-lymphocytes (P=0.032).

**Table 3:** The relationship between clinicopathological characteristics and total tumour cell pp65 expression in patients with invasive ductal breast cancer (n=376)

	low(n=74)	high(n=302)	*P*
Age (≤50/ >50 years)	21/53	87/215	0.942
Size (≤20/ 21-50/ >50 mm)	40/31/2	173/122/7	0.682
Grade (I / II / III)	18/35/21	49/11/135	**0.010**
Involved lymph node (negative/positive)	37/37	159/138	0.339
ER status (no/yes)	18/56	115/187	**0.023**
PR status (no/yes)	38/36	165/137	0.612
Her2 status (no/ yes)	62/9	238/63	0.075
Molecular subtype (Luminal A/Luminal B/triple negative/HER2 enriched)	39/18/11/3	126/61/69/41	**0.005**
Tumour necrosis (low/high)	32/40	127/169	0.813
Lymph vessel invasion (no/yes)	51/23	194/107	0.470
Blood vessel invasion (no/yes)	65/9	264/37	0.976
Klintrup–Mäkinen grade (weak/strong)	60/12	203/93	**0.013**
CD68+ (low/moderate/high)	20/32/21	75/108/118	0.206
CD4+ (low/moderate/high)	34/17/22	117/66/118	0.145
CD8+ (low/moderate/high)	18/31/24	81/108/112	0.840
CD138+ (low/moderate/high)	47/9/17	154/40/107	**0.032**
Tumour stroma percentage (low/high)	46/27	174/125	0.453
Tumour budding (low/high)	47/27	194/107	0.880
Locoregional treatment (lumpectomy+radiotherapy/mastectomy +radiotherapy)	24/50	112/190	0.456
Systemic treatment (hormonal/hormonal+ chemotherapy/chemotherapy/ none)	40/19/11/3	151/60/72/14	0.244
Recurrence (no recurrence/recurrence)	65/7	219/82	**0.001**

Bold indicates a signicant association. Number of patients with missing data is not displayed, when values do not give a sum of 100% this is due to data being unavailable.

When total tumour cell p-p65 was entered into a multivariate model using a backwards conditional method with clinicopathological parameters, total tumour cell p-p65 expression was independently associated with disease-free survival (HR 1.25(1.03-1.53), P=0.024) (Table 2).

### Does IKKβ silencing impact on breast cancer cell phenotype?

In the current study we report that activation of the canonical NF-κB pathway, measured by nuclear p-p65 expression and total tumour cell p-p65 expression, is associated with reduced cancer-specific survival. Therefore the next aim was to establish if inhibition of the canonical NF-κB pathway could offer a possible therapeutic option. MCF7 cells were chosen to represent ER positive breast tumours and MDA-MB-231 were chosen to represent ER negative breast tumours.

**Figure 3:**
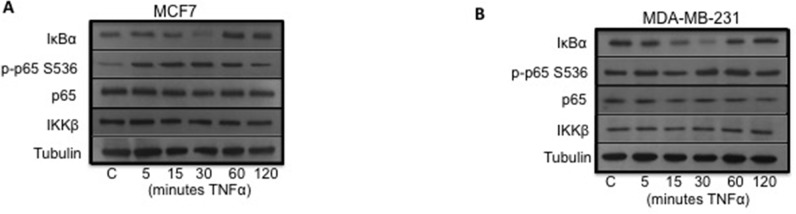
**(A)** shows Western blot and plots for MCF7 cells stimulated with TNFα. **(B)** shows Western blot and plots for MDA-MB-231 cells stimulated with TNFα.

It was first established that the canonical NF-κB pathway was inducible in both cell lines. This was achieved by treating the cells with TNFα, which is known to stimulate the canonical pathway. In MCF7, ER positive breast cancer cells, TNFα exposure induced IκBα degradation and phosphorylation of p65 in a time dependent manner (Figure 3A). Following TNFα exposure, IκBα expression was reduced compared to control at 15 minutes and 30 minutes and phosphorylation of p65 (p-p65) was increased compared to control at 15 minutes and 30 minutes. Levels of p65 and IKKβ were unchanged by TNFα exposure.

In MDA-MB-231, ER negative breast cancer cells, TNFα exposure induced IκBα degradation but to a lesser extent than that observed in the MCF7 cells and no increase in phosphorylation of p65 (p-p65) was observed (Figure 3B). Following TNFα exposure, IκBα expression was reduced compared to control at 15 minutes and 30 minutes. Levels of p65 and IKKβ were unchanged by TNFα exposure.

Having established that the canonical NF-κB pathway was active in both cell types, and inducible to a much greater extent in MCF7 cells, the next aim of the study was to assess the impact on cell viability by inhibiting the pathway via silencing of IKKβ.

**Figure 4:**
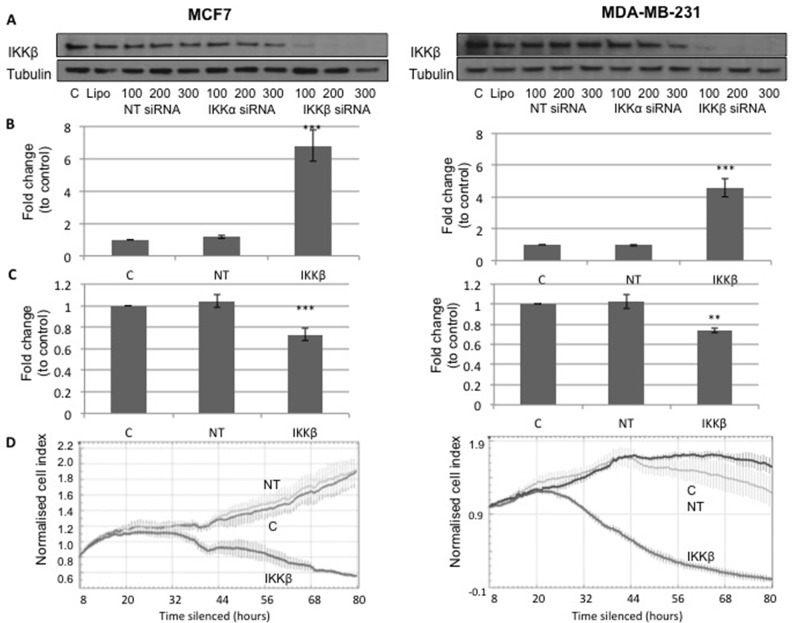
**(A)** shows Western blot for IKKβ and Tubulin expression in MCF7 ER positive cells, C is untreated cells, Lipo is cells treated only with lipofectamine, NT siRNA is cells treated with non-targeting siRNA, IKKα siRNA is cells treated with siRNA for IKKα and IKKβ siRNA is cells treated with siRNA for IKKβ. Western blot shows that only siRNA for IKKβ lowers IKKβ expression. **(B)** shows plots for fold change in apoptosis levels in MCF7 cells and MDA-MB-231 cells treated with lipofectamine (C) non-targeting siRNA (NT) and IKKβ siRNA. **(C)** shows plots for fold change in cell viability levels in MCF-7 cells and MDA-MB 231 cells treated with lipofectamine (C), non-targeting siRNA (NT) and IKKβ siRNA. **(D)** shows xCELLigence cell index plots for MCF7 cells and MDA-MB 231 cells treated with lipofectamine (C), non-targeting siRNA (NT) and IKKβ siRNA.

MCF7 and MDA-MB-231 cells were transfected with 200nM non-targeting (NT) siRNA or 200nM IKKβ siRNA, and 48 hours post treatment an apoptosis assay or cell viability assay was performed (Figure 4A). A significant increase in apoptosis was observed in cells treated with siRNA to silence expression of IKKβ compared to the NT control (MCF7 cells, P<1.0×10^−6^ and MDA-MB-231 cells, P=2×10^−4^, Figure 4B) and a decrease in cell viability was observed in cells treated with siRNA to silence expression of IKKβ compared to the NT control (MCF7 cells, P=4.6×10^−5^ and MDA-MB-231, P=0.002, Figure 4C). Cell viability was also measured using the xCELLigence machine, and cell index graphs following silencing in MCF7 and MDA-MB-231 cells (Figure 4D) plotted. Both cell types treated with siRNA to silence expression of IKKβ had reduced cell viability compared to both untreated control (C) and NT control (Figure 4D).

## DISCUSSION

In the present study expression of key members of the canonical NF-κB pathway were investigated to establish if there was a link between the canonical NF-ĸB pathway, local inflammatory infiltrate and cancer-specific survival in a cohort of patients with primary operable invasive ductal breast cancer. Phosphorylation of p65 at serine 536 (p-p65) was employed as a marker of activation of the canonical NF-κB pathway as it is important for transcriptional activation of the NF-ĸB dimer [26, 27]. High expression of p-p65 in the cytoplasm exhibited a non-significant trend towards a decrease in cancer-specific survival and high nuclear p-p65 was significantly associated with patient cancer-specific survival, recurrence and tumour grade. However no associations were made with nuclear p-p65 and inflammatory infiltrate, suggesting that this pathway is not responsible for regulating the local inflammatory response, but perhaps acting via an alternative mechanism such as proliferation.

Of additional interest was the observation that nuclear p-p65 was most strongly associated with cancer-specific survival in ER positive, Luminal B tumours. There are four main subtypes of breast cancer that are considered clinically relevant (Luminal A, Luminal B, HER2 enriched and triple negative). All subtypes have different molecular profiles and varied responses to endocrine therapy, HER2 targeted therapy and chemotherapy [3]. Differences in the biology of these subtypes are reflected in the results from this study, as activation of the NF-κB pathway has diverging roles in the different subtypes. The present study demonstrates that the association of nuclear p-p65 expression with cancer-specific survival observed in the full cohort is negated in patients with ER positive Luminal A tumours but potentiated in patients with ER positive Luminal B tumours, indicating that this association is independent of ER status. This observation adds to the growing body of evidence that Luminal A and Luminal B breast cancers should be considered as different diseases and may require separate therapeutic approaches. Currently both these patient groups receive endocrine therapy, with Luminal B patients having a worse prognosis, which is associated with an increased rate of endocrine resistance. The increased rate of endocrine resistance has previously been attributed to the presence of HER2 in this subtype, providing an alternative-signalling pathway to escape the inhibitory effects of endocrine therapy. However, not all patients with Luminal B disease exhibit overexpression of HER2 suggesting that the increased rate in endocrine recurrence in Luminal B disease might have been not be solely attributed to HER2 and might be due to an alternative mechanism in HER2 negative cases. Luminal B tumours are categorised as ER or PR positive tumours with either a high proliferation rate (Ki67 >14%) or HER2 positivity. To investigate if the increased rate of recurrence observed in Luminal B patients in the current study can be fully attributed to HER2 dependency and not NF-κB pathway activation, patients with Luminal B tumours were further divided into those with HER2 positive tumours or high Ki67. The relationship with p-p65 and cancer-specific survival was maintained in highly proliferative tumours and not in those with HER2 positive tumours. Only very small numbers were available for analysis, so this observation should be confirmed in a larger cohort but does suggest that in the high Ki67 subgroup, activation of the NF-κB pathway may also play a role in the increased rate of recurrence attributed to development of endocrine resistance. This in itself is not a novel observation as the NF-κB pathway has previously been associated with development of endocrine resistance [28–30] and NF-κB activity is enhanced in Tamoxifen resistant MCF7 cells compared to MCF7 Tamoxifen sensitive cells [28]. However, this is the first study to provide evidence suggesting that the NF-κB pathway may promote recurrence in Luminal B highly proliferative tumours, offering an alternative therapeutic strategy for patients with HER2 negative Luminal B disease. This approach may be employed in combination with endocrine treatment to delay development of endocrine resistance or as a therapeutic option following development of endocrine resistance.

To further investigate this hypothesis mechanistic studies examining the effect and most efficient method of suppressing activation of NF-κB pathways are required. The present study investigated the impact of a reduction in IKKβ expression by siRNA transfection on MCF7 cell viability, using various methods, including assessment of apoptosis using an ELISA, viability using a WST-1 assay, real time growth and viability using xCELLigence cell index. All modes of assessment demonstrated that reduction in IKKβ expression resulted in a decrease of cell viability. These results taken together with our clinical cohort results suggest that it may be beneficial to block NF-κB activity in ER positive Luminal B patients in combination with endocrine therapy or following development of endocrine resistance.

Although the effect we observed was potentiated in patients with ER positive, Luminal B disease, a trend towards significance was also observed in patients with ER, PR, HER2 negative (triple negative) disease but not ER negative, HER2 enriched disease. Although the trend did not reach significance in patients with triple negative disease, 10 year cancer-specific survival was stratified from 82% to 53%. Significance was possibly not met due to the study being underpowered to observe differences in this subtype. This is also in concordance with the *in vitro* work in the current study as high levels of phosphorylation of p65 was observed in ER negative MDA-MB-231 cells and suppression of the pathway via a reduction in IKKβ expression by siRNA resulted in decreased cell viability. This is in line with the literature where high levels of NF-ĸB activity have been reported in ER-negative tumours [28].

In addition to investigating associations between nuclear p-p65 expression and patient outcome measures, a cumulative prognostic score combining both cytoplasmic and nuclear p-p65 was examined. Using this cumulative prognostic score the current study observed that total cell expression was a stronger predictor of cancer-specific survival compared to nuclear alone and was independently associated with cancer-specific survival when combined with clinico-pathological parameters. In addition to the increased prognostic power observed for total tumour cell p-p65 compared to nuclear p-p65, total tumour cell p-p65 was associated with more measurements of the local inflammatory response. Nuclear p-p65 was only associated with tumour grade, tumour stroma percentage, recurrence and cancer-specific survival, in comparison to total tumour cell p-p65 being associated with tumour grade, ER status, molecular subtype, Klintrup-Makinen grade, CD138+ cells, recurrence and cancer-specific survival. This suggests that examining total cell p-p65 expression results in a more accurate, robust measurement of activated NF-ĸB in the tumour cell prior to or following nuclear translocation. Therefore total tumour cell p-p65 may be employed as a possible prognostic marker, or predictive marker for therapies targeting NF-ĸB. When stratified by molecular subtype, although not reaching significance, a trend was observed between reduced cancer-specific survival and total tumour cell p-p65 in the Luminal B and triple negative subtypes, in line with that observed for nuclear p-p65.

In summary, results from the present study demonstrate a significant role of the canonical NF-κB pathway in the progression of breast cancer, which appears to be greater in Luminal B and triple negative subtypes. Selective novel compounds targeting NF-ĸB pathway may offer a promising therapeutic approach. Furthermore, clinical trials should be designed incorporating predictive biomarkers to ensure that only subtypes known to respond to NF-κB intervention would receive treatment to maximise benefit and minimise unwanted toxicities.

## MATERIALS AND METHODS

### Patient cohorts

The TMA included tumour tissue samples from 376 breast cancer patients presenting with invasive ductal breast cancer between 1995 and 1998 in the West of Scotland (at Glasgow Royal Infirmary, Glasgow Western Infirmary and Stobhill Hospital). Clinico-pathological data available included age, tumour grade, tumour size, lymph node status, therapy, ER, PR and HER2 status and Ki67 proliferation index. Information on inflammatory infiltrate and tumour microenvironment had previously been established for the cohort [18–23]. H&Es slides were employed to assess tumour stroma percentage (TSP) and Klintrup-Makinen as previously reported [19–21]. Lymph and blood vessel invasion (LVI and BVI, respectively) were assessed, using IHC staining with the lymphatic endothelial marker D2-40 and vascular endothelial marker Factor VIII as previously described [31].

Tissue microarrays (TMAs) made from formalin-fixed paraffin-embedded tissue (FFPE) blocks which were retrieved from pathology archives were already available for this retrospective study. Tumour rich areas selected for construction of the TMA were identified by a consultant pathologist. Ethical approval for the use of this tissue was granted by the research Ethics Committee of the North Glasgow University Hospitals NHS Trust (NHS GG&C rec no 10/50704/60). In accordance with REMARK criteria, the markers examined, study objectives and hypothesis was described. Patient clinicopathological characteristics have been described, specimen characteristics provided, IHC methods and antibody specificity confirmed. Biomarkers have been shown in relation to prognostic variables and univariate and multivariate analysis applied.

### Immunohistochemistry

Immunohistochemistry (IHC) was performed to assess protein levels of members of the NF-κB pathways; IKKβ and phosphorylated p65 subunit at serine 536 of NF-κB (p-p65). TMAs were cut into 2.5μm thick sections, tissue was dewaxed by immersion in xylene and rehydrated through a series of graded alcohols. Heat induced antigen retrieval was performed in a solution of either citrate buffer pH6 (IKKβ) or Tris EDTA buffer pH9 (p-p65). Tissue was then incubated in 3% hydrogen peroxide before non-specific binding was blocked by incubation in either 5% normal horse serum solution (Vector Laboratories; IKKβ) or 1x caesin solution (Vector Laboratories; p-p65,). Slides were then incubated in primary antibody overnight at 4°C (IKKβ, p-p65). Antibodies were diluted to optimal concentration in antibody diluent (Dako). Primary antibodies for anti-IKKβ was ab32135, Abcam at 1:500 and for anti p-p65 was ab28856, Abcam at 1:25. Staining was developed using EnVision™ (Dako) and 3,3′-diaminobenzidine (DAB; Vector Laboratories). Harris Haematoxylin counterstaining was performed and tissue was dehydrated and mounted using DPX.

Stained TMA sections were scanned using a Hamamatsu NanoZoomer (Welwyn Garden City, Hertfordshire, UK) at x20 magnification and visualization was carried out using Slidepath Digital Image Hub, version 4.0.1 (Slidepath, Leica Biosystems, Milton Keynes, UK). Protein expression was assessed using the weighted histoscore method [LB], with a second independent observer [JE] scoring 10% of cores and the interclass correlation coefficient (ICCC) calculated to ensure no observer bias [24]. P-p65 cytoplasmic and nuclear expression within the cytoplasm and nucleus were calculated separately.

### Cell culture

MCF7 and MDA-MB-231 breast cancer cells were cultured in Dulbecco’s Modified Eagle Medium (DMEM) (Life Technologies) with 10% Fetal Bovine Serum (FBS) (Sigma-Aldrich), 10 Units/ml Penicillin/Streptomycin (Life Technologies) and 1x GlutaMAX™ (Life Technologies) in 5% CO_2_ at 37°C.

### Ligand exposure

Cells were seeded in 12 well plates at 1×10^5^ cells per well and once 70% confluence was reached, cells were rendered quiescent by serum deprivation for 24 hours before being stimulated by exposure to 20ng/ml TNFα (Sigma-Aldrich) at a range of time points (5 minutes, 15 minutes, 30 minutes, 60 minutes and 120 minutes).

### Western blotting

At end of incubations/exposure to agents, cells were lysed in pre-heated Laemmli’s sample buffer, dispered by repeating passing through a 21G needle and heated at 100°C for 5 min prior to analysis. SDS-Polyacrylamide Gel Electrophoresis (SDS-PAGE) was performed to separate proteins, which were then transferred to nitrocellulose membranes by electrophoretic blotting in wet conditions. Non-specific binding then blocked in 3% (w/v) BSA in NaTT buffer and membranes were incubated overnight, either at room temperature or 4°C, in primary antibody specific to the target protein diluted to optimal concentration in NaTT buffer containing 0.3% (w/v) BSA. Antibodies and dilutions used were as follows: p65 (1:10000, sc-8008, Santa Cruz), p-p65 (1:1000, #3031, Cell Signaling), IκBα (1:7500, #1242, Cell Signaling), IKKβ (1:2500, ab32135, Abcam) and β-tubulin (at 1:5000, ab21058, Abcam).

Membranes were incubated in secondary HRP-conjugated antibody (either rabbit or mouse, depending on primary antibody) diluted 1:10000. Enhanced chemiluminescence (ECL) reagent was used to detect presence of the antibody and X-ray films developed using an X-OMAT machine (Kodak) or the G:Box imaging system (SynGene).

### Silencing

Cells were transfected with ON-TARGETplus siRNA (Thermo Scientific) targeting IKKβ (*IKBKB*, #J-003503-13). In order to confirm the observed effects were due to the loss of IKKβ and not the transfection procedure, cells were treated with all reagents (lipofectamine® diluted in Opti-MEM®) but no siRNA as a control (C) and were transfected with non-targeting sequence as a control (non-targeting #1, #D-001810-01-20) (NT). IKKβ siRNA at 100 nM for all, 200 nm and 300 nm was delivered to the cells using lipofectamine® RNAiMAX (Life Technologies) diluted in Opti-MEM® (Life Technologies) and media was replaced with normal DMEM containing 10% (v/v) FCS after 6 hours. Following 48 hours silencing, a Western blot was performed to establish the extent of silencing (Figure 4), extent of IKKα silencing was also investigated [32]. From these results a concentration of 200nM IKKβ siRNA was chosen as the concentrations used in all future experiments for both cell lines.

### Apoptosis and cell viability assays

After knockdown of mRNA expression, cells were plated for apoptosis or cell viability assays. Apoptosis levels were assessed using a Cell Death Detection enzyme-linked immunosorbent assay kit (Roche) and for cell viability using the water-soluble tetrazolium salt (WST-1) reagent (Roche). Cells were seeded in a 96 well plate at a density of 5×10^3^ cells/well in 100μL of standard culture medium. Once a confluency of 60-70% was reached cells were exposed to siRNA for 48 hours and the assays then performed following manufacturer’s instructions. Assays were performed in triplicate and error bars representing standard deviation added to graphs. Fold change was compared to control.

### Cell viability via xCELLigence

The xCELLigence machine (ACEA Biosciences, San Diego) was used to display cell growth and viability in real time following silencing of IKKβ. With this method, measurements are continuously sent to the computer, allowing for real time growth curves to be plotted using “Cell Index” which represents the number and viability of the cells [25]. Cells were seeded in a 96 well *E-plate™* (ACEA Biosciences, San Diego) at 3×10^3^ cells/well with 200μl of media in each well, grown for two days to ensure log phase of growth before treatment with siRNA and after 72 hours graphs showing cell index over time were drawn.

### Statistical analysis

Statistics was performed using IBM SPSS version 21. Kaplan-Meier curves were constructed for cancer-specific survival, the log rank test was employed to compare high and low expression. Hazard ratios were calculated using Cox regression with 95% confidence intervals. Cox regression multivariate analysis was also performed with the inclusion of known predictive factors. Inter-relationships between variables were assessed using contingency tables with the chi-squared test for trend as appropriate. Values of P<0.05 were considered statistically significant.

Statistical analysis for apoptosis and cell viability assays was performed using a two-way ANOVA with Bonferroni correction and Dunnett’s test. P values were considered significant if P<0.05 and highly significant if P<0.001.

## SUPPLEMENTARY FIGURES


