# Supplementary material for: The relationship between members of the canonical NF-κB pathway, components of tumour microenvironment and survival in patients with invasive ductal breast cancer
Source: Oncotarget. 2017 Mar 9;8(20):33002–13. doi: 10.18632/oncotarget.16031 (PMC5464845; doi:10.18632/oncotarget.16031)
Supplement: Supplementary file 1 [file oncotarget-08-33002-s001.pdf]

The relationship between members of the canonical NF-κB pathway, components of tumour microenvironment and survival in patients with invasive ductal breast cancer

SUPPLEMENTARY FIGURES

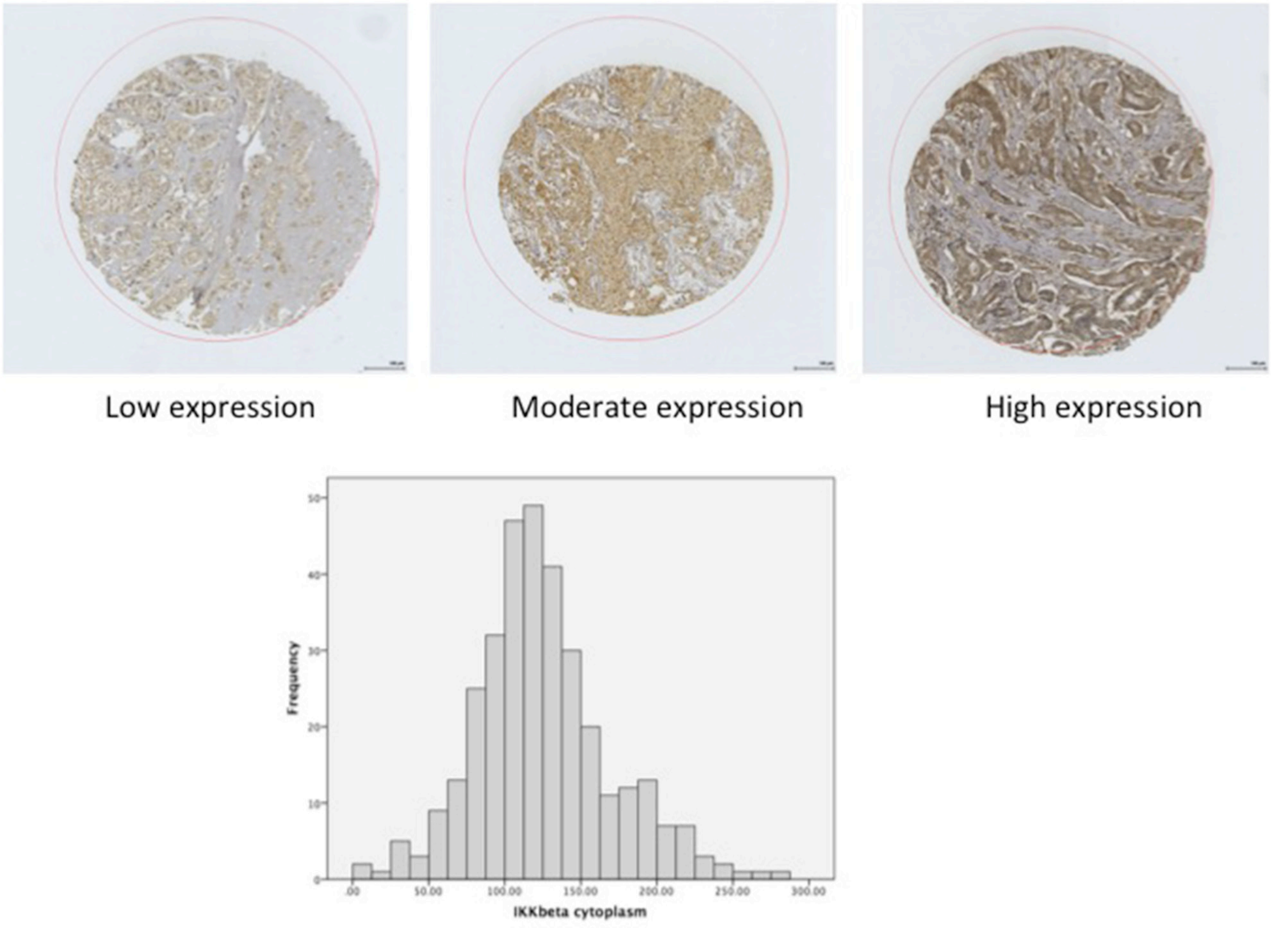

Supplementary Figure 1: Displays microphotographs illustrating low, moderate and high staining for IKKβ, with a histogram of the distribution of cytoplasmic IKKβ protein expression. Magnification x400.

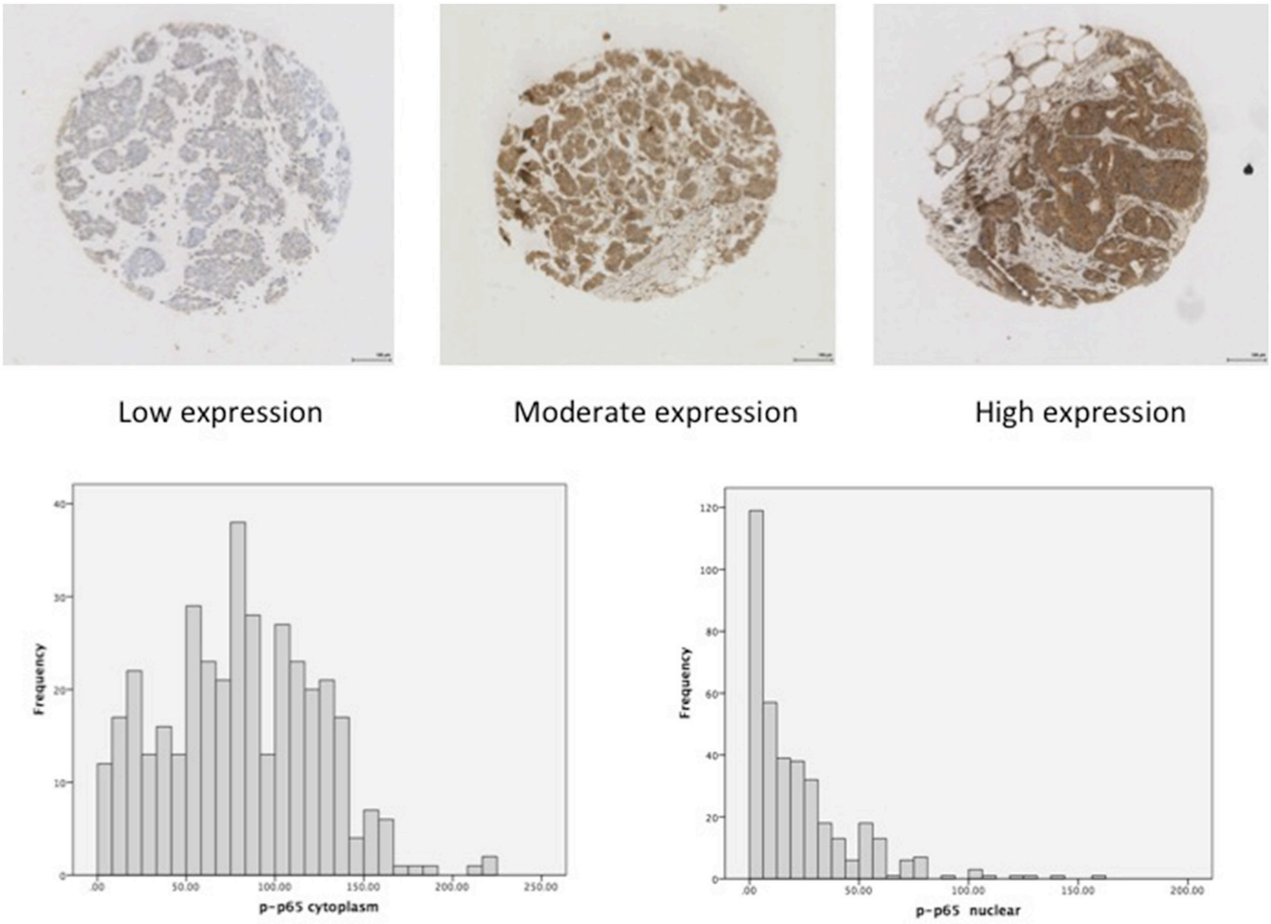

Supplementary Figure 2: Displays microphotographs illustrating low, moderate and high staining for p-p65, with histograms of the distribution of both cytoplasmic and nuclear p-p65 protein expression. Magnification x400.
